# Tuberculosis Verrucosa Cutis Developing Over A Keloid: A Rare Presentation

**DOI:** 10.4103/2006-8808.73620

**Published:** 2010

**Authors:** Sanjay Kala, Chayanika Pantola, Asha Agarwal

**Affiliations:** *Department of General, Surgery, G.S.V.M. Medical College, Kanpur, UP, India*; 1 *Department of Pathology, G.S.V.M. Medical College, Kanpur, UP, India*

**Keywords:** Cutaneous tuberculosis, keloid, tuberculosis verrucosa cutis

## Abstract

Cutaneous tuberculosis can present as either primary or secondary infection, or it can be associated with systemic tuberculosis. It can present with unusual clinical and histological features causing delay in diagnosis and treatment. Tuberculosis verrucosa cutis occurs as a single verrucous lesion over exposed areas of the body along with inflammatory borders and discharging sinus. Here, we are presenting a rare case of tuberculosis verrucosa cutis developing over a keloid. There is no report of such case in literature so far.

## INTRODUCTION

Tuberculosis was until recently considered to be a diminishing clinical problem in industrialized nations, while remaining a dominant public health problem in resource-poor countries. However, there is a global resurgence of tuberculosis because of a combination of factors including immigration from endemic countries, increased movement of refugees, the HIV pandemic, and poverty. As a result, cutaneous tuberculosis remains a clinical and diagnostic problem.[1] It has various clinical and morphological forms depending on the mode of entry and whether it is a primary or secondary infection.[2] Tuberculosis verrucoa cutis represents an inoculated exogenous infection of the skin in persons with a degree of immunity, i.e., previous exposure to tuberculosis. It is usually observed as a single verrucous plaque with inflammatory borders. Here, we are presenting a case of tuberculosis verrucosa cutis developing over a keloid; such a case has not been reported in the literature.

## CASE REPORT

**Figure 1:**
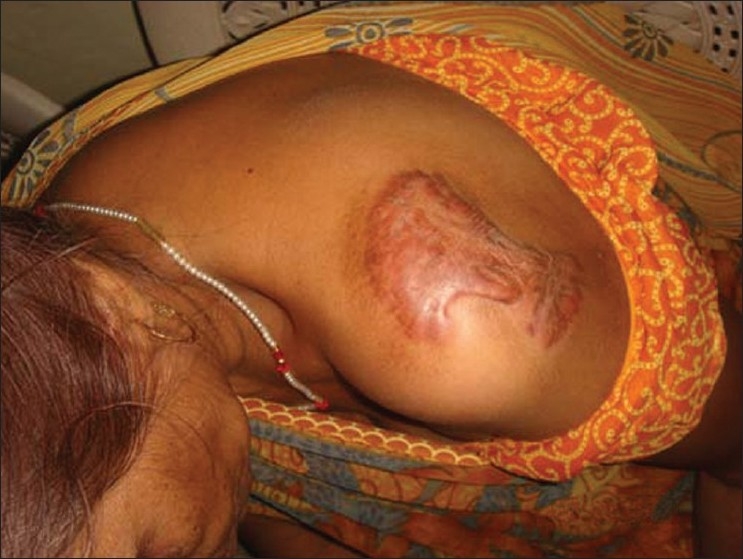
Patient having keloid over back

**Figure 2:**
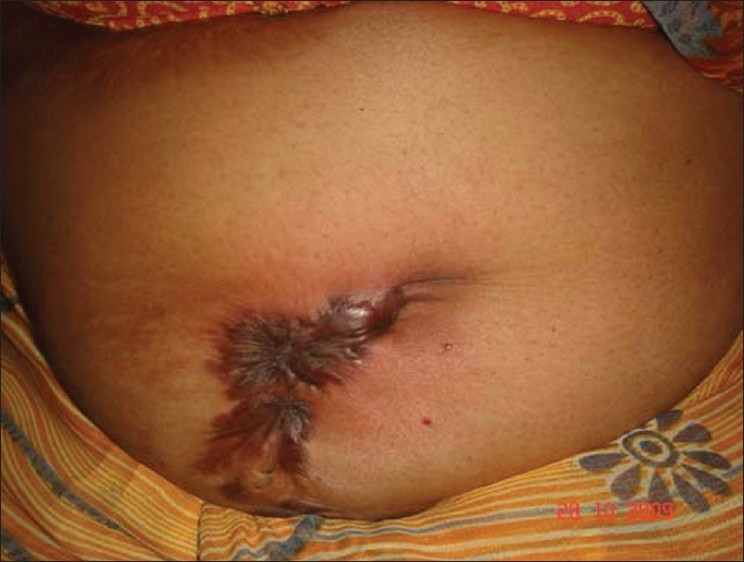
Keloid over anterior abdominal wall having tuberculosis verrucosa cutis

**Figure 3:**
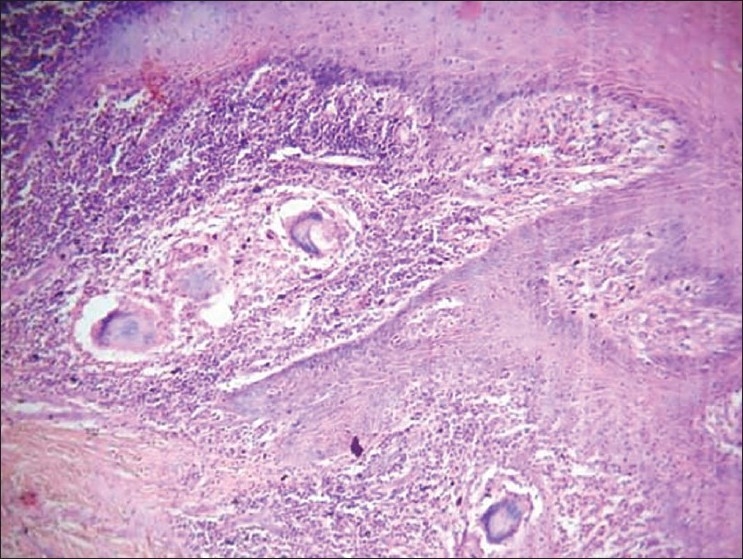
Tubercular granuloma showing giant cells just beneath the epidermis (H and E, ×400)

A 70-year-old otherwise healthy lady presented with multiple keloid over the back, shoulder, and anterior abdominal wall for the last 40–50 years [Figures 1 and 2]. Five years prior to presentation the anterior abdominal wall keloid had sloughed off with a discharging sinus developing over it. Fine Needle A spiration cytology was done, which showed granuloma formation and the smears were also positive for Acid Fast B acilli with ZN staining. Histological examination revealed a well defined granuloma just beneath the epidermis [Figure 3]. Her chest X-ray was normal and no other significant findings were found on clinical examination and investigations. A diagnosis of tuberculosis verrucosa cutis developing over a keloid was made.

## DISCUSSION

Cutaneous tuberculosis can present with an unusual clinical and histological features causing delay in diagnosis.[3] Sometime they may be associated with systemic tuberculosis, thus a thorough clinical examination in all cutaneous tuberculosis is necessary to exclude systemic tuberculosis.[4]

Tuberculosis verrucosa cutis represents an inoculated exogenous infection of the skin presented as single verrucous lesion. The verrucous surface exhibits fissures from which pus often can be expressed. Overall the most common sites are hands and in children, the knees, buttocks, and thighs. Multiplicity of the skin lesion and systemic involvement may be due to poor health and haematogenous dissemination.

Microscopically, histological pictures represent hyperkeratosis and acanthosis. Beneath the epidermis there is usually an acute inflammatory infiltrate. Abscess formation may be observed in the upper dermis. In mid-dermis tubercular granulomas, moderate amount of necrosis is usually present. Tuberculous bacilli are more numerous in the disease and can be demonstrated by ZN staining. Other forms of cutaneous tuberculosis are lupus vulgaris, scrofuloderma, tuberculosis cutis orificialis, etc. Disseminated form of cutaneous tuberculosis are not uncommon specially lupus vulgaris.[5] Review of literature have shown report of lupus[6] as well as giant sized lupus[7] developing on keloid, but tuberculosis verrucosa cutis developing over a keloid is a rare presentation, as in our case. Thus, knowledge of this possibility and good clinical acumen is necessary to make a diagnosis.
